# Unexpected inhibition of the lipid kinase PIKfyve reveals an epistatic role for p38 MAPKs in endolysosomal fission and volume control

**DOI:** 10.1038/s41419-024-06423-0

**Published:** 2024-01-22

**Authors:** Daric J. Wible, Zalak Parikh, Eun Jeong Cho, Miao-Der Chen, Collene R. Jeter, Somshuvra Mukhopadhyay, Kevin N. Dalby, Shankar Varadarajan, Shawn B. Bratton

**Affiliations:** 1 Department of Epigenetics & Molecular Carcinogenesis, The University of Texas MD Anderson Cancer Center, Houston, TX 77054 USA; 2 Targeted Therapeutic Drug Discovery and Development Program, Division of Chemical Biology & Medicinal Chemistry, College of Pharmacy, The University of Texas at Austin, Austin, TX 78712 USA; 3 Division of Pharmacology & Toxicology, College of Pharmacy, The University of Texas at Austin, Austin, TX 78712 USA; 4 Institute of Translational Medicine, University of Liverpool, Liverpool, L69 3BX UK

**Keywords:** Kinases, Macroautophagy, Targeted therapies, Target identification

## Abstract

p38 mitogen-activated protein kinases (MAPKs) participate in autophagic signaling; and previous reports suggest that pyridinyl imidazole p38 MAPK inhibitors, including SB203580 and SB202190, induce cell death in some cancer cell-types through unrestrained autophagy. Subsequent studies, however, have suggested that the associated cytoplasmic vacuolation resulted from off-target inhibition of an unidentified enzyme. Herein, we report that SB203580-induced vacuolation is rapid, reversible, and relies on the class III phosphatidylinositol 3-kinase (PIK3C3) complex and the production of phosphatidylinositol 3-phosphate [PI(3)P] but not on autophagy per se. Rather, vacuolation resulted from the accumulation of Rab7 on late endosome and lysosome (LEL) membranes, combined with an osmotic imbalance that triggered severe swelling in these organelles. Inhibition of PIKfyve, the lipid kinase that converts PI(3)P to PI(3,5)P2 on LEL membranes, produced a similar phenotype in cells; therefore, we performed in vitro kinase assays and discovered that both SB203580 and SB202190 directly inhibited recombinant PIKfyve. Cancer cells treated with either drug likewise displayed significant reductions in the endogenous levels of PI(3,5)P2. Despite these results, SB203580-induced vacuolation was not entirely due to off-target inhibition of PIKfyve, as a drug-resistant p38α mutant suppressed vacuolation; and combined genetic deletion of both p38α and p38β dramatically sensitized cells to established PIKfyve inhibitors, including YM201636 and apilimod. The rate of vacuole dissolution (i.e., LEL fission), following the removal of apilimod, was also significantly reduced in cells treated with BIRB-796, a structurally unrelated p38 MAPK inhibitor. Thus, our studies indicate that pyridinyl imidazole p38 MAPK inhibitors induce cytoplasmic vacuolation through the combined inhibition of both PIKfyve and p38 MAPKs, and more generally, that p38 MAPKs act epistatically to PIKfyve, most likely to promote LEL fission.

## Introduction

p38 mitogen-activated protein kinases (MAPKs) have reported roles in membrane trafficking, including during endocytosis and autophagy. During early endocytosis, for example, p38 MAPKs increase the rates of receptor internalization mediated by the Rab GTPase, Rab5, through phosphorylation and activation of GDP dissociation inhibitor α (GDIα) and early endosome antigen 1 (EEA1) [1–4]. During autophagy, isolation membranes originate from *trans*-Golgi network (TGN)-derived vesicles containing the lipid scramblase, ATG9A [5, 6]; and p38 MAPKs regulate ATG9A through phospho-dependent sequestration of p38IP, an ATG9A interactor that influences its trafficking [7]. ATG9A vesicles are then recruited to pre-autophagosomal structures (PAS) at endoplasmic reticulum (ER) exit sites, where they serve as the initial membrane source for the growth of phagophores [8, 9].

Class III phosphatidylinositol 3-kinase (PIK3C3) complexes, composed of VPS34, Beclin 1, and VSP15, selectively associate with endosomes or phagophores, depending upon their interactions with specific adapter proteins, UVRAG or ATG14L, respectively, and catalyze the production of phosphatidylinositol 3-phosphate [PI(3)P] [10, 11]. Maturation of early endosomes into late endosomes (LEs) is marked by the conversion of PI(3)P into phosphatidylinositol 3,5-bisphosphate [PI(3,5)P2] by the lipid kinase, PIKfyve (also known as PIP5K3), concomitant with the replacement of Rab5 for Rab7 [12–14]. PI(3,5)P2 on LE membranes then recruit various factors involved in the sorting of degradative cargo and intralumenal vesicle formation [15, 16].

The precise mechanisms that mediate the transfer of LE contents to lysosomes remain somewhat controversial, but LEs likely fuse with lysosomes to produce endolysosomal “hybrid” organelles [17]. PI(3)P^+^ autophagosomes also converge with the endosomal pathway at this stage by fusing with LEs and lysosomes in a Rab7-dependent manner [18, 19]. Following digestion of cargo within hybrid organelles, LEs and lysosomes are then recovered as a result of fission through a process that involves PI(3,5)P2-dependent release of Ca^2+^ from lysosomal lumens and lysosomal acidification via the V-ATPase [17, 20, 21].

Notably, previous reports have suggested that the p38 MAPK inhibitors, SB203580 and SB202190, induce cytoplasmic vacuolation and cell death, particularly in colorectal cancer cells, through unrestrained autophagy, raising the possibility that p38 MAPK inhibitors might be clinically useful in the treatment of certain cancers [22, 23]. However, the role(s) for p38 MAPKs in autophagy are conflicting with numerous reports suggesting that p38 MAPKs can either suppress or promote autophagy, apparently depending upon context [7, 22–28]; and indeed, subsequent studies have argued that vacuolation induced by these drugs is due to the inhibition of an unidentified target rather than p38 MAPKs [29–31].

Herein, we report that pyridinyl imidazole p38 MAPK inhibitors induce profound cytoplasmic vacuolation in many cancer cell-types independently of autophagy. Interestingly, however, while inhibition of p38 MAPKs was required for vacuolation, it was insufficient to do so, implying that simultaneous inhibition of yet another enzyme was necessary. Using a phenotypic drug discovery approach, we subsequently identified PIKfyve as a direct target of both SB203580 and SB202190. PIKfyve inhibition resulted in a substantial decrease in PI(3,5)P2 levels and LEL swelling in treated cells, but simultaneous inhibition of p38 MAPKs was also required to promote cytoplasmic vacuolation through suppression of LEL fission. Thus, in summary, we have identified PIKfyve as a direct target of pyridinyl imidazole p38 MAPK inhibitors, and we have uncovered a previously unrecognized role for p38 MAPKs in regulating endolysosomal fission epistatic to PIKfyve.

## Results

### SB203580 and SB202190 induce cytoplasmic vacuolation through combined inhibition of p38 MAPKs and an “off-target” enzyme

**Fig. 1:**
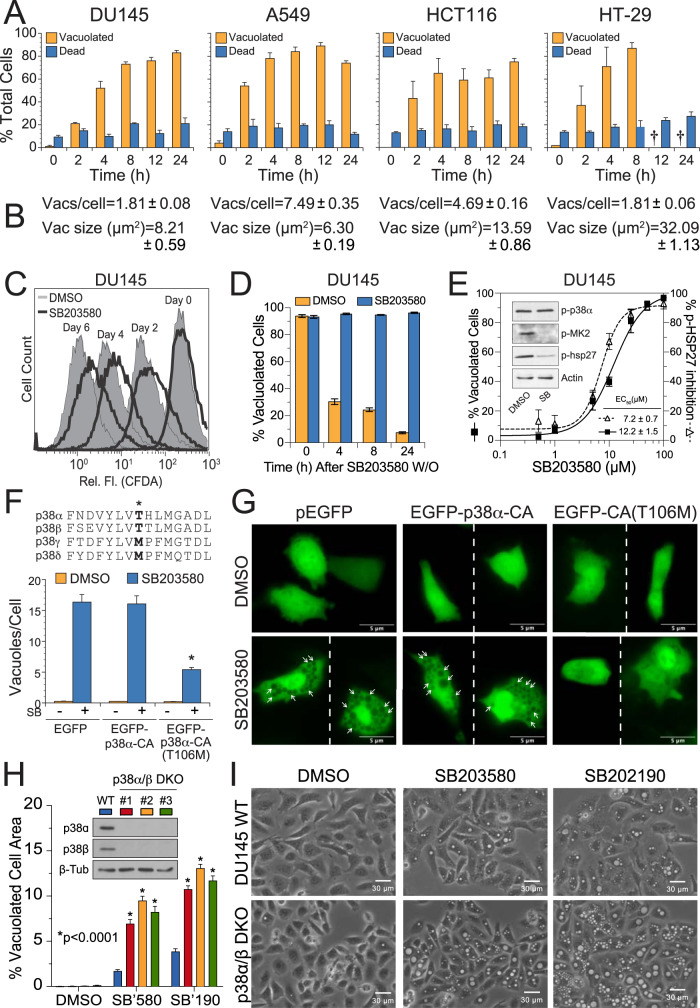
Pyridinyl imidazole p38 MAPK inhibitors induce cytoplasmic vacuolation. **A**, **B** Various cancer cell lines were treated with DMSO (control) or SB203580 (50 µM) for 2–24 h and assessed for vacuolation and cell death by phase-contrast microscopy and flow cytometry (annexin V-propidium iodide staining), respectively (†, not determined; see also Supplementary Fig. S1A). At 24 h, <3% of DMSO-treated cells were vacuolated and cell death values (%) were: DU145 (9 ± 0.4), A549 (12.8 ± 0.8), HCT116 (15.0 ± 1.6), HT-29 (10.2 ± 1.2). The number of vacuoles per cell and the average vacuole size (µm^2^) was determined using ImageJ analysis of phase-contrast images. **C** DU145 cells were stained with CFDA-SE, exposed to DMSO (shaded peaks) or SB203580 (empty peaks), and monitored for changes in cell proliferation, as described in the methods. **D** DU145 cells were treated with SB203580 (50 µM) for 24 h and then washed and replaced with fresh media ± SB203580 for 4–24 h. At each time point following the washout, cells were examined for vacuolation by phase-contrast microscopy (see also Supplementary Fig. S1C). **E** DU145 prostate cancer cells were treated with increasing concentrations of the pharmacological p38 MAPK inhibitor, SB203580 (0–100 µM), for 24 h and examined for signs of vacuolation by phase-contrast microscopy (200×). Inset: SB203580 (50 µM) inhibited p38-dependent sequential phosphorylation of MK2 and HSP27. Concentration-dependent inhibition of HSP27 phosphorylation by SB203580 (0–100 µM) was also determined by western blotting (see Supplementary Fig. S1D, E) with individual bands scanned, quantified with ImageJ software, and plotted as the percent of p-HSP27 inhibited. **F**, **G** DU145 cells were transiently transfected with expression plasmids encoding EGFP, constitutively active (D176A/F327S) p38α (EGFP-p38α-CA), or p38α-CA containing an additional mutation to the gatekeeper residue (T106M) that renders p38α resistant to SB203580 [EGFP-p38α-CA (T106M)]. EGFP-positive cells were then evaluated by fluorescence microscopy for the number of vacuoles present per cell. **H**, **I** p38α and p38β were deleted from DU145 cells using CRISPR-Cas9; and three p38 DKO clones were exposed to SB203580 (50 µM) or SB202190 (50 µM) and evaluated for vacuolation by phase-contrast microscopy using ImageJ analysis software.

In studies begun many years ago, we observed that the pyridinyl imidazole p38 MAPK inhibitors, SB203580 and SB202190, induced profound cytoplasmic vacuolation in multiple cancer cell lines including prostate (DU145, PC-3), lung (A549), colon (HCT116, HT-29), and cervical cancers (HeLa), as well as immortalized mouse embryonic fibroblasts (MEFs) (Fig. 1A, B and Supplementary Fig. S1A). In marked contrast, pharmacological inhibitors of other stress and growth-related kinases, including c-Jun N-terminal kinases (JNKs, SP600125), extracellular signal-regulated kinases (ERKs, PD98059), mammalian target of rapamycin (mTOR, rapamycin), and phosphoinositide 3-kinases (PI3Ks, LY294002) failed to induce comparable levels of vacuolation (Supplementary Fig. S1B). SB203580-induced vacuolation did not lead to a substantial increase in cell death across multiple cell types, at least over a 24 h time span (Fig. 1A), and caused only a moderate reduction in DU145 cell proliferation during several days in culture, as determined by CFDA labeling (Fig. 1C). Vacuolation was also entirely reversible with most cells returning to normal within 8 h following the washout of drug (Fig. 1D and Supplementary Fig. S1C).

SB203580 inhibited the p38 MAPK pathway, as indicated by a concentration-dependent decrease in the sequential phosphorylation of MAP kinase-activated protein kinase 2 (MK2) and heat shock protein 27 (HSP27) (Fig. 1E and Supplementary Fig. S1D), and pathway inhibition correlated strongly with an increase in cellular vacuolation (Supplementary Fig. S1E, *R*^2^ = 0.82). SB202190 likewise inhibited p38 MAPK-dependent phosphorylation of HSP27 (Supplementary Fig. S1F). SB203580 inhibits the activities of p38α and p38β isoforms, but not p38δ and p38γ, due to the presence of a threonine (rather than a methionine) “gatekeeper” residue in their ATP-binding pockets (Fig. 1F, inset) [32, 33]. Therefore, to confirm that selective inhibition of p38α/β MAPKs by SB203580 was responsible for (or at least contributed to) cytoplasmic vacuolation, we performed rescue experiments wherein cells were transfected with an EGFP-tagged, constitutively active mutant of p38α (D176A/F327S; termed p38α-CA) [34], possessing either the wild-type threonine or a mutated methionine residue [p38α-CA(T106M)] [35]. Upon exposure of cells to SB203580, those transfected with either EGFP or EGFP-p38α-CA underwent normal vacuolation, whereas those transfected with EGFP-p38α-CA(T106M) were resistant to SB203580-induced vacuolation (Fig. 1F, G).

Thus, selective inhibition of p38α/β MAPKs (hereafter referred to simply as p38 MAPKs) by SB203580 appeared to be responsible for the observed vacuolation. However, when we utilized CRISPR-Cas9 to delete both p38α and p38β from DU145 cells, the complete absence of p38 MAPK proteins failed to trigger vacuolation (Fig. 1H, I). These p38 MAPK double knockout (DKO) cells nevertheless were even more sensitive to SB203580 and SB202190-induced vacuolation (Fig. 1H, I). Thus, collectively, the data indicated that inhibition of p38 MAPKs by SB203580 was required but insufficient to induce cytoplasmic vacuolation—simultaneous inhibition of an unknown enzyme was also essential. We therefore began the process of searching for the critical “off-target” of pyridinyl imidazole inhibitors using a phenotypic drug discovery approach.

### SB203580 does not induce autophagy but requires PIK3C3 activity to induce vacuolation

**Fig. 2:**
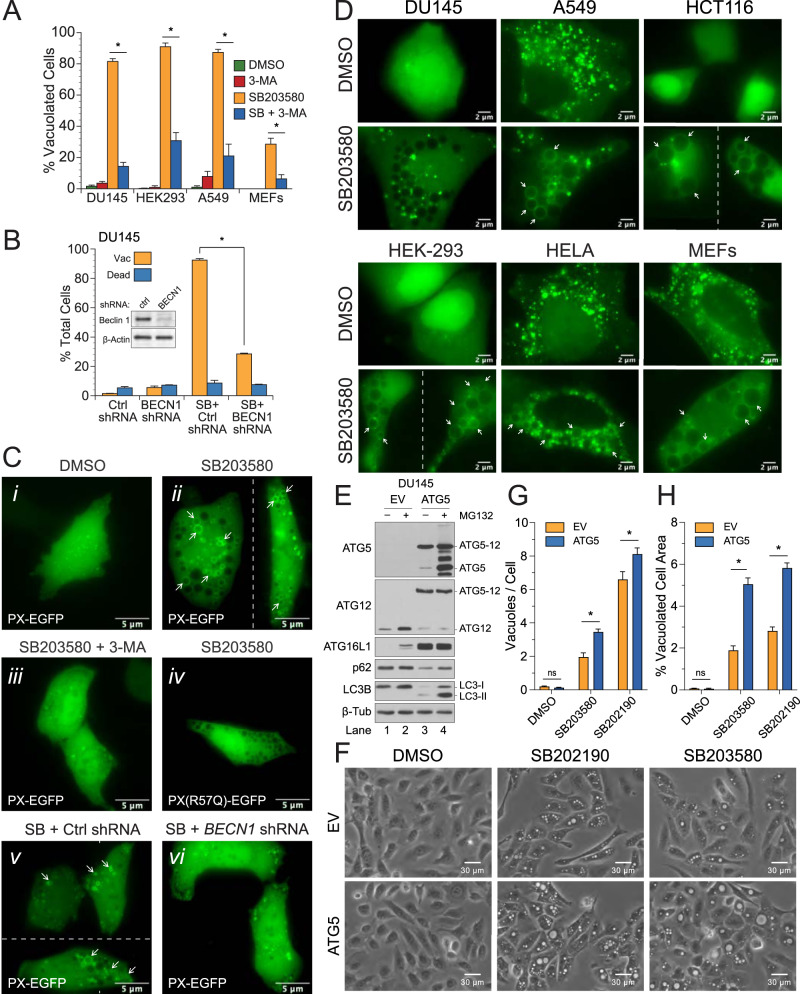
SB203580 does not stimulate autophagy but does induce vacuolation in a PIK3C3-dependent manner and incorporates pre-existing autophagosome membranes. **A** DU145, HEK293, A549, and MEFs were exposed to SB203580 (50 µM) ± the class III PI3 kinase inhibitor, 3-MA (5 mM), and examined for vacuolation. For each cell-type, asterisks indicate that 3-MA was significantly different from the SB treatment alone (one-way ANOVA with Student–Neumann–Keuls post hoc analysis; *p* < 0.05). **B** DU145 cells were stably transfected with scrambled control or *beclin 1* shRNA plasmids, treated with DMSO (control) or SB203580 (50 µM), and examined for vacuolation and cell death. Inset: Beclin 1 expression levels were determined by immunoblotting. **C** DU145 cells were transiently transfected with the PI(3)P probe, PX-EGFP, or the binding mutant R57Q, and subsequently treated with (i) DMSO or (ii–iv) SB203580 ± 3-MA for 24 h. Similarly, (v) control and (vi) Beclin 1-depleted cells were also transfected with PX-EGFP and treated with SB203580 (50 µM) for 24 h and examined by fluorescence microscopy. **D** Various cancer cell lines and MEFs were transiently transfected with EGFP-LC3 and treated with SB203580 (50 µM) for 24 h. Some but not all LC3-labeled vacuoles are indicated with arrows. **E**–**H** DU145 were reconstituted with ATG5 to restore basal autophagy; exposed to SB203580 (50 µM) or SB202190 (50 µM) for 24 h; and evaluated by phase-contrast microscopy for vacuolation.

Previous reports suggested that vacuolation induced by SB203580 and SB202190 resulted from autophagic cell death that was otherwise suppressed by p38 MAPKs [22, 23]. While SB203580 induced very little cell death in our hands (Fig. 1A), inhibition of the autophagy-essential class III PI3-kinase PIK3C3 (also known as VPS34) with 3-methyladenine (3-MA) [36] did dramatically inhibit vacuolation in all cell lines tested (Fig. 2A), as did stable shRNA knockdown of the PIK3C3 accessory protein/regulator Beclin 1 in DU145 cells (Fig. 2B). To verify that 3-MA effectively inhibited PIK3C3 activity, we generated an EGFP construct fused to a single PX domain of p40Phox (PX-EGFP), as well as a PI(3)P-binding mutant (PX-R57Q-EGFP) [37]. Following exposure to SB203580, many of the small to medium-sized vacuoles were clearly labeled with PX-EGFP but not with PX-R57Q-EGFP (Fig. 2C, images ii and iv). Cotreatment with 3-MA or knockdown of Beclin 1, on the other hand, inhibited the formation of PI(3)P and produced a diffuse cytoplasmic staining pattern, similar to that observed in control cells (Fig. 2C, images i, iii, and vi). Thus, SB203580 induced the formation of cytoplasmic vacuoles, many of which were enriched in PI(3)P; and inhibition of PIK3C3 with 3-MA or knockdown of Beclin 1 inhibited both PI(3)P production and vacuole formation.

In parallel experiments, to confirm the formation of autophagosomes and determine if the large vacuoles arose out of an increase in autophagy, we transfected cells with EGFP-tagged microtubule-associated protein 1 light chain 3 (EGFP-LC3) and examined them for evidence of autophagosome formation, as indicated by the accumulation of EGFP-LC3 puncta [38]. No puncta were observed in DU145, HCT116, or HEK293 control cells, whereas numerous puncta were observed in A549, HELA, and MEF control cells, suggesting that the latter possessed higher rates of basal autophagy (Fig. 2D, upper panels). Following treatment with SB203580, vacuoles in all cell lines were decorated with EGFP-LC3 with the notable exception of DU145 cells (Fig. 2D, lower panels). Cell lines that displayed the highest levels of basal autophagy, prior to treatment with SB203580, likewise exhibited the greatest degree of vacuole labeling following treatment, implying that EGFP-LC3 from pre-existing autophagosomes was incorporated into developing vacuoles following fusion (Fig. 2D).

We were puzzled at the time, however, as to why the SB203580-induced vacuoles in DU145 cells were not labeled with EGFP-LC3 (Fig. 2D, lower panel), so we examined DU145 cells using transmission electron microscopy (TEM). To our surprise, all SB203580-induced vacuoles were found to be single-bilayer rather than the double-bilayer membrane structures characteristic of autophagosomes (Supplementary Fig. S2A). Larger vacuoles (>2 μm diameter) were mostly empty (Supplementary Fig. S2A, image ii), whereas smaller vacuoles, which were not readily identifiable by phase-contrast microscopy, contained undigested vesicles and cytoplasmic material reminiscent of multivesicular bodies (MVBs) (Supplementary Fig. S2A, images iii–iv). We would later discover that DU145 cells were naturally deficient in ATG5, due to a donor splice site gene mutation, and thus were incapable of undergoing canonical autophagy (Fig. 2E, lanes 1 and 2) [39]. Long-lived protein degradation (LLPD) assays confirmed an absence of autophagy in DU145 cells in response to SB203580 or starvation (Supplementary Fig. S2B). Notably, however, SB203580 also failed to stimulate an increase in LLPD in “autophagy-competent” A549, HCT116, or HT-29 cells (Supplementary Fig. S2B).

Even though SB203580 did not induce autophagy (Fig. 2D and Supplementary Fig. S2), the fact that EGFP-LC3 readily labeled vacuoles in autophagy-competent cell types (Fig. 2D) led us to question whether autophagosomes, resulting from basal autophagy, might still contribute membrane to the expanding vacuole. To evaluate this possibility, we reconstituted DU145 cells with ATG5 (Fig. 2E, lanes 3 and 4) and found that restoration of autophagy slightly increased the number of vacuoles formed per cell, following treatment with SB203580 (Fig. 2F, G), but more substantially increased the size of those vacuoles (Fig. 2F, H). Thus, in summary, SB203580 stimulated vacuolation in a PIK3C3-dependent manner but did not induce canonical autophagy per se, although pre-existing autophagosomes, resulting from basal autophagy, could contribute to vacuole expansion.

### SB203580 stimulates Rab7-dependent enlargement of late endosomes and lysosomes

**Fig. 3:**
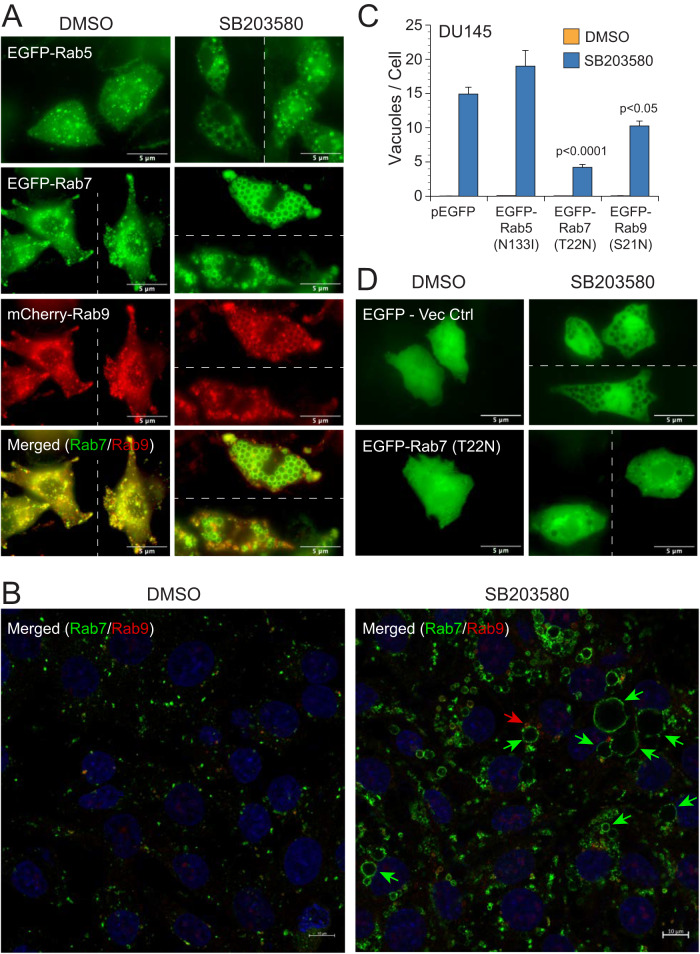
SB203580-induced vacuolation results from Rab7-dependent fusion of LEs and lysosomes. **A** DU145 cells were co-transfected with various combinations of EGFP or mCherry-labeled Rab5, Rab7, and/or Rab9. The cells were then treated with DMSO (control) or SB203580 (50 µM) for 24 h and examined for colocalization with the vacuoles and/or one another. **B** DU145 cells, treated for 24 h with DMSO (control) or SB203580 (50 µM), were evaluated by immunofluorescence microscopy using antibodies to endogenous Rab7 (green) and Rab9 (red) (see also Supplementary Fig. S3B). **C**, **D** DU145 cells were similarly transfected with either EGFP (empty vector) or dominant-negative Rab7(T22N). Cells were then exposed to DMSO or SB203580 (50 µM), and the number of vacuoles was counted in at least 50 cells by fluorescence microscopy. Each experiment was performed in triplicate, and each data point represents mean ± SEM.

TEM micrographs strongly suggested that the vacuoles induced by SB203580 were enlarged MVBs/LEs (Supplementary Fig. S2A); however, in order to confirm and further characterize these vacuoles, we transfected DU145 cells with fluorescent fusion proteins of various early endosome (Rab5), LE (Rab7, Rab9) and lysosome (LAMP1) markers, alone or in combination. As anticipated, all vacuoles were extensively decorated with Rab7, with a smaller fraction co-labeling with Rab9 and/or LAMP1 (Fig. 3A and Supplementary Fig. S3A). Staining with antibodies to endogenous Rab7 and Rab9 proteins confirmed that SB203580-induced vacuoles were heavily decorated with Rab7, whereas only a few, much smaller, vacuoles were labeled with Rab9 (Fig. 3B and Supplementary Fig. S3B). More interestingly, vacuolation was substantially reduced following the expression of a GDP-locked dominant-negative mutant of Rab7(T22N) and, to a much lesser extent, Rab9(S21N), but not following the expression of Rab5(N133I) (Fig. 3C, D and Supplementary Fig. S3C). Conversely, vacuolation was dramatically enhanced by a constitutively active mutant of Rab7(Q67L), so much so that accurately counting vacuoles was impractical (Supplementary Fig. S3C). Thus, collectively, the results strongly indicated that Rab7 accumulated on SB203580-induced vacuoles and mediated the enlargement of LEs and lysosomes (hereafter referred to as LELs) (Fig. 3 and Supplementary Fig. S3).

### SB203580 disrupts the osmotic balance and function of LELs

**Fig. 4:**
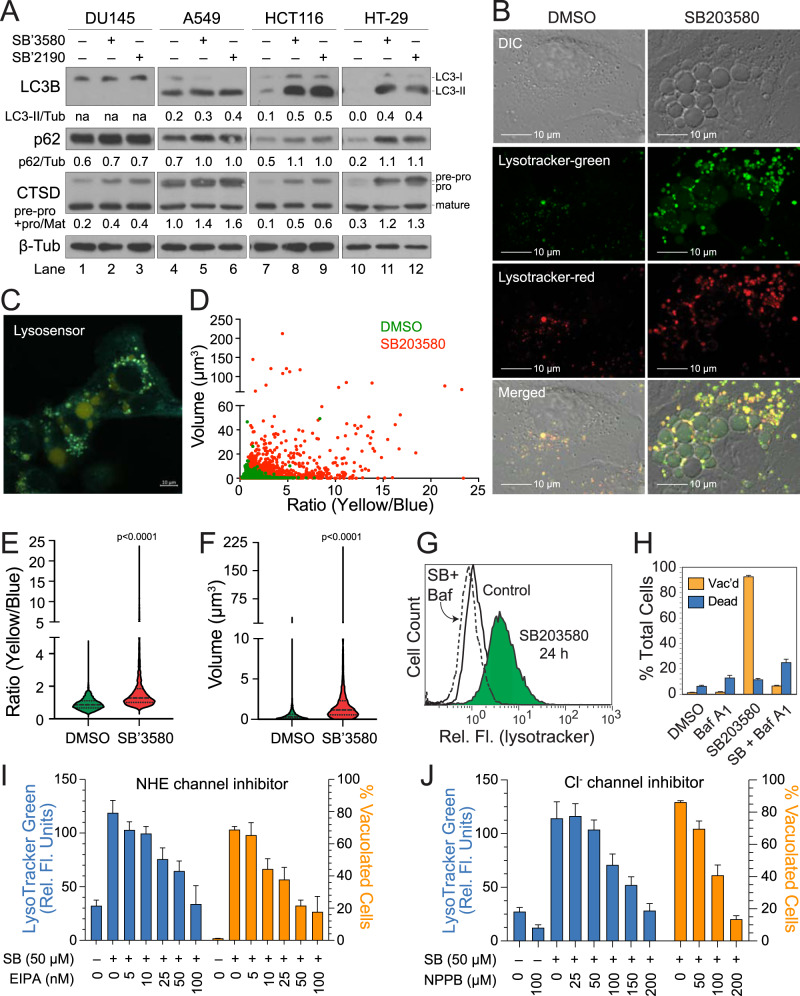
SB203580 induces LEL swelling and defects in cathepsin processing and protein degradation. **A** DU145, A549, HCT116, and HT-29 cells were exposed to SB203580 (50 µM) or SB202190 (50 µM) and western blotted for LC3B, p62, CTSD, or β-tubulin. Band intensities were determined by ImageJ; and the LC3B-II/β-tubulin, p62/β-tubulin, and pre-pro/mature CTSD ratios are listed below the indicated immunoblots. **B** DU145 cells were exposed to DMSO or SB203580 (50 µM) for 24 h, labeled with LysoTracker^TM^ Green and Red, and evaluated by confocal microscopy or flow cytometry. **C**–**F** DU145 cells were exposed to DMSO or SB203580 (50 µM) for 24 h, loaded with LysoSensor^TM^ Yellow/Blue DND-160 (2 µM), and evaluated 10 min later by confocal microscopy using ratiometric imaging (Ex = 360 nm; Em = 404–456 nm and 510–641 nm). LysoSensor^TM^-labeled vesicles/vacuoles were evaluated for volume and acidity (yellow/blue ratio), and plotted alone or against one another. **G**–**J** DU145 cells were treated with SB203580 (50 µM) for 24 h, in the presence or absence of a V-ATPase inhibitor (Bafilomycin A1, 125 nM), a Na+-H+ exchanger inhibitor (ethylisopropyl amiloride, i.e., EIPA, 5–100 nM), or a chloride channel inhibitor (5-nitro-2-(3-phenylpropyl-amino)benzoic acid, i.e., NPPB, 25–200 µM). The cells were then assayed for acidic compartments by flow cytometry (LysoTracker^TM^ Green) and evaluated for vacuole formation by phase-contrast microscopy.

p38 MAPK inhibitors were initially reported to induce autophagy in large part due to an apparent increase in LC3B lipidation [22], which we too observed in autophagy-competent cells, including HCT116 and HT-29 cells (Fig. 4A). However, SB203580 failed to stimulate LLPD in these same cell lines (Supplementary Fig. S2B), suggesting that the increase in lipidated LC3B (LC3-II) might result instead from a decrease in autophagic flux. Consistent with this interpretation, treatment of cells with SB203580 or SB202190 also decreased the pre-pro processing of cathepsin D (CTSD) and suppressed the degradation of p62 (Fig. 4A). Since cathepsin activity is pH-dependent, we first questioned whether SB203580 treatment resulted in LEL alkalinization but subsequently discovered that SB203580 stimulated the formation of numerous highly acidic vesicles that readily stained with both LysoTracker^TM^ Green and Red dyes (Fig. 4B). The more acidic vesicles were not visible by differential interference contrast (DIC) imaging but did appear to aggregate and coalesce around the much larger translucent and more weakly stained vacuoles (Fig. 4B). This suggested that fusion of the smaller acidic vacuoles might have given rise to the much larger vacuoles [40], whereupon dilution of the dye resulted in decreased fluorescence. Therefore, we instead utilized LysoSensor^TM^ Yellow/Blue DND-160, a ratiometric dye that is largely immune to dilution effects and produces blue or yellow fluorescence at more neutral or acidic pH, respectively. Following SB203580 treatment, we found that the larger vacuoles were, in fact, even more acidified than their smaller counterparts, implying that a relationship existed between the size and acidity of the vacuoles (Fig. 4C–F). Therefore, we next pretreated cells with Bafilomycin A_1_ (Baf A), a potent inhibitor of the V-ATPase that alkalinizes LELs and inhibits their fusion with one another and autophagosomes [41–43]. As predicted, Baf A profoundly suppressed (and even reversed) both SB203580-induced cellular acidification and vacuolation (Fig. 4G, H and Supplementary Fig. S4A, B).

While acidification of vesicles is required for membrane fusion, we speculated that the larger translucent vacuoles might also result from increased swelling, perhaps due to increased turgor pressure (Fig. 4B and Supplementary Fig. S2A). Indeed, following washout of SB203580, LysoTracker^TM^ Green staining of cells rapidly returned to normal (Supplementary Fig. S4C); and the vacuoles underwent collapse (Supplementary Fig. S4D). This led us to question whether an initial acidification of smaller vesicles facilitated membrane fusion but eventually led to an influx of water that resulted in severe vacuole (i.e., LEL) swelling. Consistent with this interpretation, incubation of cells in hypertonic medium containing sorbitol dramatically reduced SB203580-induced vacuolation (Supplementary Fig. S4E). Mechanistically, several ion transporters, including Na^+^/H^+^ exchangers (NHEs) and Cl^-^/H^+^ exchangers (CLCs), exchange protons for sodium or chloride ions, resulting in an influx of salt into the lumens of LELs (Supplementary Fig. S4G). With this in mind, we pretreated cells with the NHE inhibitors, EIPA or Zonaporide, or the chloride channel inhibitors, NPPB or DIDS, and observed concentration-dependent decreases in LysoTracker^TM^ Green staining and/or vacuole formation (Fig. 4I, J and Supplementary Fig. S4H, I). These effects appeared to be specific, since inhibitors of the CFTR chloride channel or Na^+^/Ca^2+^ exchangers had no significant effects (data not shown). Thus, the profound effects of both Baf A and EIPA on SB203580-induced vacuolation were most likely attributable to an inhibition in the proton-driven influx of NaCl and water into the lumens of LELs. Severe swelling of these LELs likely hindered both cathepsin processing/activity and degradation of any delivered cargo due to increased volume of the enlarged compartment.

### SB203580 and SB202190 stimulate vacuolation through off-target inhibition of PIKfyve

At this point in our phenotypic drug discovery approach, we had determined that vacuoles induced by SB203580 and SB202190 were enlarged LELs that accumulated PI(3)P (Fig. 2 and Supplementary Fig. S2), were decorated with excessive Rab7 (Fig. 3 and Supplementary Fig. S3), and were swollen due to an osmotic imbalance that likely resulted in decreased LEL fission (Fig. 4 and Supplementary Fig. S4). We noted with interest that recently developed inhibitors of the lipid kinase PIKfyve, which catalyzes the conversion of PI(3)P to PI(3,5)P2, induced a strikingly similar cytoplasmic vacuolation [44–48]. Moreover, PIKfyve is known to positively regulate the activities of at least two classes of ion channels: ‘transient receptor potential’ (TRP) channels, including TRPML1 (also known as MCOLN1), and ‘two pore channels’ (TPCs) (Supplementary Fig. S4G) [49–55]. Thus, PIKfyve inhibition and the subsequent decrease in PI(3,5)P2 is reportedly associated with dysregulation of calcium and sodium efflux, LEL membrane potential, swelling, and ultimately results in decreased intralumenal invagination and LEL fission [16, 48, 56, 57].

**Fig. 5:**
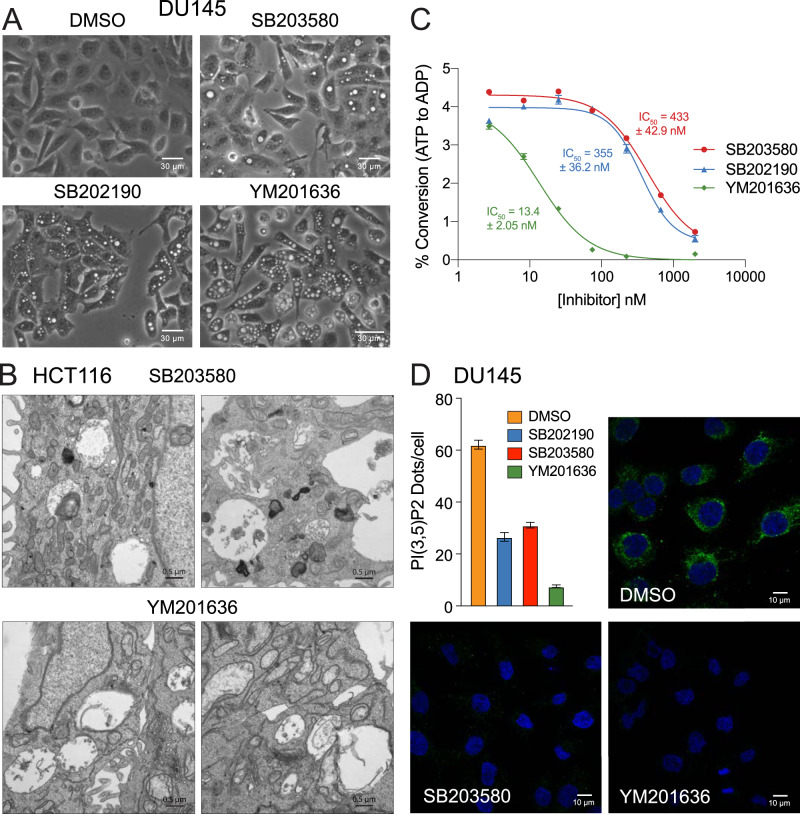
Pyridinyl imidazole p38 MAPK inhibitors are direct inhibitors of the lipid kinase PIKfyve. **A** DU145 cells were treated with DMSO, SB203580 (50 µM), SB202190 (50 µM), or YM201636 (1 µM) for 24 h and then evaluated for vacuolation by phase-contrast microscopy. **B** HCT116 cells were treated with SB203580 (50 µM) or YM201636 (1 µM) for 24 h and analyzed by transmission electron microscopy (14,000×, bars = 500 nm). **C** Recombinant PIKfyve was incubated with PI(3)P, phosphatidylserine, and ATP, in the presence of increasing concentrations of SB203580, SB202190, or YM201636, and assayed for ATP hydrolysis using ADP Glo luminescence reagent, as described in the methods. **D** DU145 cells were treated with SB202190 (50 µM), SB203580 (50 µM), or YM201636 (1 µM) for 24 h and evaluated for PI(3,5)P2 levels by immunofluorescence microscopy, as described in the methods.

Given these striking similarities, we compared the effects of the PIKfyve inhibitor, YM201636, with SB203580 and SB202190 and found that all three stimulated the formation of vacuoles that appeared indistinguishable from one another (Fig. 5A, B). This led us to question whether SB203580 and SB202190 might induce vacuolation by directly inhibiting PIKfyve and reducing the cellular levels of PI(3,5)P2. Therefore, we incubated recombinant PIKfyve with increasing concentrations of SB203580, SB202190, or YM201636 and measured the conversion of ATP-to-ADP in the presence of the substrate PI(3)P (Fig. 5C). While less potent than YM201636, both SB203580 and SB202190 inhibited PIKfyve activity in vitro with IC_50_ values of 433 ± 42.9 nM and 355 ± 36.2 nM, respectively (Fig. 5C). Moreover, when we evaluated cells treated with SB203580, SB202190, or YM201636, we observed a corresponding reduction in the levels of PI(3,5)P2 (Fig. 5D), as determined by immunofluorescence staining [31]. Thus, the pyridinyl imidazole p38 MAPK inhibitors induce cytoplasmic vacuolation in cells, at least in part, through “off-target” inhibition of PIKfyve, thereby reducing the levels of PI(3,5)P2 necessary for normal LEL function.

### p38 MAPKs suppress vacuolation triggered by PIKfyve inhibition

**Fig. 6:**
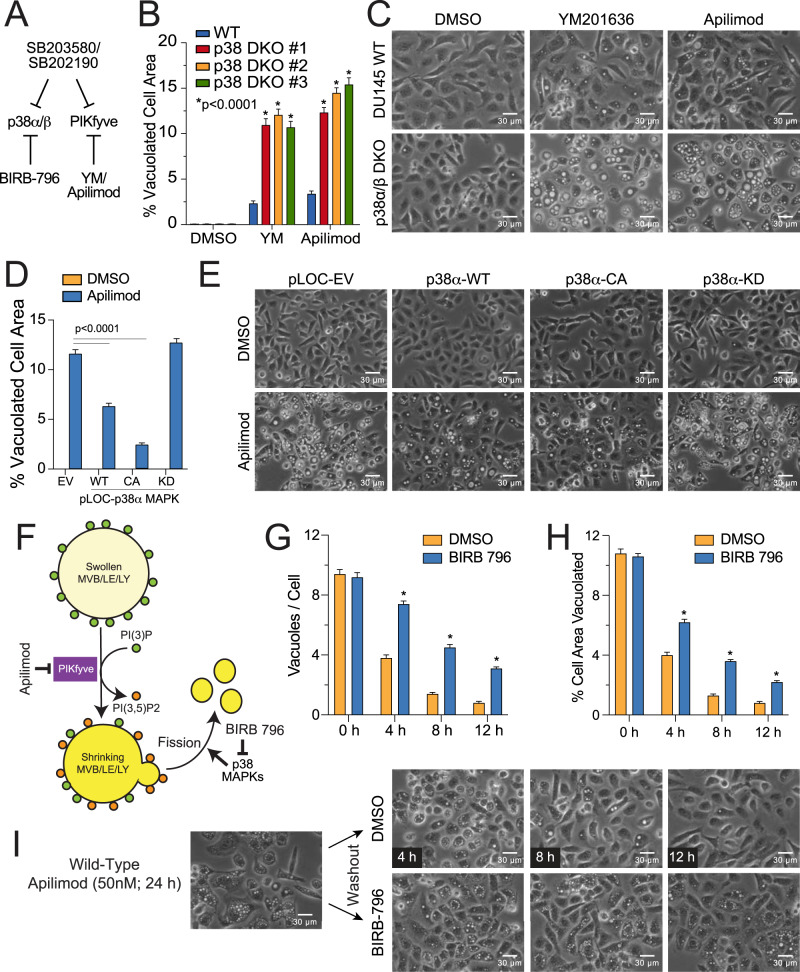
Loss or inhibition of p38 MAPKs sensitizes cells to vacuolation induced by PIKfyve inhibitors. **A** Model to explain how SB203580 and SB202190 induce vacuolation through combined inhibition of p38 MAPKs and PIKfyve. **B**, **C** p38 DKO clones were exposed to YM201636 (500 nM) or apilimod (20 nM) for 24 h and evaluated for vacuolation by phase-contrast microscopy using ImageJ analysis software. **D**, **E** p38 DKO cells were stably reconstituted with a vector control, or wild-type (WT), constitutively active (CA), or kinase dead (KD) versions of p38α, exposed to apilimod (20 nM), and evaluated for vacuolation by phase-contrast microscopy using ImageJ analysis software. **F** Model for epistatic relationship between PIKfyve and p38 MAPKs. **G**–**I** Wild-type DU145 cells were treated with apilimod (50 nM) for 24 h, after which apilimod was removed and BIRB-796 (50 µM) was added to selectively inhibit p38 MAPKs during the “washout” phase. Vacuolation was assessed by phase-contrast microscopy over time (0–12 h) using ImageJ software to determine both the number of vacuoles per cell and percent cell area vacuolated. Inhibition of p38 MAPKs delays the resolution of vacuoles, implicating a role for p38 MAPKs in promoting LEL fission.

Despite successfully identifying PIKfyve as an “off-target” of SB203580 and SB202190, it remained notable that a constitutively active p38α, containing the drug-resistant T106M gatekeeper mutant, significantly rescued cells from SB203580-induced vacuolation (Fig. 1F, G). This strongly suggested that p38 MAPKs could suppress vacuolation induced by PIKfyve inhibition and that SB203580 stimulated vacuolation through simultaneous inhibition of both PIKfyve and p38 MAPKs (Fig. 6A). If so, we predicted that p38 DKO cells should be particularly sensitive to vacuolation induced by selective PIKfyve inhibitors; and indeed, vacuolation was dramatically more severe in p38 DKO cells following treatment with YM201636 or apilimod, with the cytoplasm of some cells being almost entirely occupied by a single very large vacuole (Fig. 6B, C). Acute pharmacological inhibition of p38 MAPK activities with BIRB-796—a structurally unrelated p38 MAPK inhibitor that did not induce vacuolation on its own—likewise exacerbated YM201636 and apilimod-induced vacuolation in wild-type DU145 cells (Supplementary Fig. S5).

Finally, when we reintroduced wild-type (WT), constitutively active (CA), or kinase dead (KD) p38α MAPKs into DKO cells, p38α-WT and particularly p38α-CA dramatically suppressed apilimod-induced vacuolation, whereas p38α-KD had no apparent effect (Fig. 6D, E), affirming that p38 MAPK activity per se was required to suppress vacuolation induced by PIKfyve inhibition. Collectively, these data led us to speculate that p38 MAPKs might play a role in promoting LEL fission (Fig. 6F). If so, we reasoned that the rate of vacuole resolution (i.e., LEL fission), following the removal of the PIKfyve inhibitor, should be reduced when p38 MAPKs are selectively inhibited. Therefore, we treated DU145 cells with apilimod to induce vacuolation, after which cells were washed and incubated with either DMSO or BIRB-796 for 0–12 h (Fig. 6G–I). Following “washout”, BIRB-796 significantly delayed the resolution of vacuoles, strongly suggesting that p38 MAPKs play a significant role in LEL fission (Fig. 6G–I). Thus, p38 MAPKs act epistatic to PIKfyve with regard to LEL fission; and pyridinyl imidazole p38 MAPK inhibitors are effective at inducing cytoplasmic vacuolation due to their combined “on-target” inhibition p38 MAPKs and “off-target” inhibition of PIKfyve.

## Discussion

In this study, we observed cytoplasmic vacuolation in cells exposed to the pyridinyl imidazole p38 MAPK inhibitors, SB203580 and SB202190; and we confirmed that p38α was an important target using a drug-resistant “gatekeeper” mutant that suppressed vacuolation (Fig. 1 and Supplementary Fig. S1). However, CRISPR-mediated deletion of both p38α and p38β from cells failed to trigger vacuolation indicating that p38 MAPK inhibition alone was required but insufficient to induce vacuolation and that SB203580 must have another target enzyme whose simultaneous inhibition was essential for vacuolation (Fig. 1). This led us to utilize a modified phenotypic drug discovery approach to identify the “off-target” of SB203580 that was responsible for vacuolation in the context of p38 MAPK inhibition [58]. During our studies, published reports suggested that vacuolation induced by SB202190 resulted from an increase in autophagic cell death [22, 23]. However, while PIK3C3 was essential for SB203580-induced vacuolation, SB203580 itself did not induce autophagy per se, at least as defined by an increase in the degradation of cellular cargo (Fig. 2 and Supplementary Fig. S2). In fact, SB203580 suppressed autophagic flux, resulting in an accumulation of LC3B-II and p62 in autophagy-competent cells (Fig. 4A). Since vacuolation might theoretically result from any number of defects in endomembrane trafficking, we sought to characterize the nature of the vacuoles and identified them as severely swollen LELs (Figs. 3 and 4 and Supplementary Figs. S2 and S4). Growth of these vacuoles was dependent on Rab7 and was driven by an osmotic imbalance in LELs, resulting from an initial hyper-acidification, followed by a subsequent influx of salt and water (Figs. 3 and 4 and Supplementary Figs. S3 and S4).

At this stage in the project, we noted that the phenotypes of SB203580 and SB202190-induced vacuoles were remarkably similar to those observed following treatment with recently developed PIKfyve inhibitors. We subsequently demonstrated that both SB203580 and SB202190 could directly inhibit PIKfyve in vitro, albeit at lower potency, and substantially reduced the production of PI(3,5)P2 in treated cells (Fig. 5). Moreover, we found that p38 DKO cells were dramatically more sensitive to vacuolation induced by selective PIKfyve inhibitors (Fig. 6). Finally, through a series of pharmacological “wash-out” experiments, involving selective PIKfyve inhibitors and the structurally unrelated p38 MAPK inhibitor, BIRB-796, we confirmed that p38 MAPKs most likely suppress vacuolation by promoting LEL fission (Fig. 6). Thus, pyridinyl imidazole p38 MAPK inhibitors, SB203580 and SB202190, induced cytoplasmic vacuolation through a combination of PIKfyve and p38 MAPK inhibition, resulting in profound LEL swelling and a reduction in LEL fission.

Our study underscores the inherent challenges of phenotypic drug discovery, particularly in instances where a compound possesses activities on different targets at distinct steps in the same pathway. However, our characterization of SB203580 has revealed an epistatic relationship between PIKfyve and p38 MAPKs that would likely have been difficult to uncover otherwise. Indeed, loss of p38 MAPKs (or inhibition of them with BIRB-796) was insufficient to induce vacuolation on its own; and complete inhibition of PIKfyve activity results in profound vacuolation that can mask the effects of p38 MAPK inhibition. It is therefore only in the context of weaker PIKfyve inhibition that p38 MAPK inhibition reveals its true impact on cytoplasmic vacuolation.

Importantly, there are at least two practical implications from our study: Firstly, considering the recent interest in the clinical use of PIKfyve inhibitors, particularly for the treatment of various cancers [47, 48, 59], and given that complete intratumoral inhibition of PIKfyve or p38 MAPK activities is unlikely in vivo, combined use of PIKfyve and p38 MAPK inhibitors may prove therapeutically useful in the future. This is particularly true, since p38 MAPK pathways are reportedly activated by PIKfyve inhibitors in cancer cells [60]. Secondly, although SB203580 and SB202190 are themselves unlikely to be utilized clinically, it is worth emphasizing from a basic science perspective that both compounds have frequently been employed to evaluate the roles of p38 MAPKs in various endomembrane trafficking processes including endocytosis and autophagy. Many of these processes are likely to be impacted by unexpected off-target inhibition of PIKfyve, so care should be exercised when using these compounds and results from previous studies may warrant reevaluation with this fact in mind.

Finally, while our phenotypic drug discovery approach to SB203580-induced vacuolation identified PIKfyve as a critical target (and helped demonstrate its epistatic relationship to p38 MAPKs), the specific downstream target(s) of p38 MAPKs that regulate LEL fission remain unknown and are the focus on ongoing work. Notably, p38 MAPKs phosphorylate the lysosomal transmembrane protein, LAMP2A, in order to promote chaperone-mediated autophagy (CMA) [61], which mediates the targeting of a specific subset of soluble proteins to lysosomes, independently of autophagomes [62]. O’Connell and Vassilev argue that p38 MAPKs, activated in response to PIKfyve inhibition, may also promote canonical autophagy (and compensate for PIKfyve defects) through increased LAMP2 phosphorylation [60]. However, if LAMP2 phosphorylation promotes autophagosome-lysosome fusion, as suggested, the increased rate of fusion would presumably result in larger (not smaller) vacuoles in the context of PIKfyve inhibition and defects in LEL fission. Moreover, autophagosome-deficient DU145 cells clearly undergo enhanced vacuolation in response to combined PIKfyve and p38 MAPK inhibition, all of which strongly suggests that relevant p38 MAPK targets, in addition to LAMP2, are likely to exist.

PIKfyve inhibition clearly disrupts normal osmotic balance in LELs and induces swelling; and a decrease in PI(3,5)P2 levels is known to reduce the activities of various ion channels, including TRPML1, TPCs, and V-ATPases in mammals and/or yeast [49–55]. Thus, it is possible that p38 MAPKs positively regulate the activities of one or more of these channels (or others), some of which possess putative phosphorylation sites, and in turn promote LEL fission by reducing swelling. Still other potential targets exist, including those proteins that constitute the putative lysosome-specific fission machinery such as WIPI, SNX, and dynamin proteins [63–65]. Given the relatively large number of conceivable targets, a phospho-proteomic analysis of wild-type and p38 MAPK-deficient (or acutely inhibited) cells, analyzed following exposure to PIKfyve inhibitors and during washout of the drug, may offer the best opportunity to identify those targets of p38 MAPKs that are critical for LEL fission.

## Materials and methods

### Reagents

SB203580, SB202190, SP600125, PD98059, LY294002, Bafilomycin A1, and rapamycin were obtained from Alexis Corp. (San Diego, CA) or Tocris Cookson (Ellisville, MO). All other chemicals were of analytical grade and purchased from Sigma-Aldrich or EMD Biosciences (Gibbstown, NJ). Antibodies to ATG5 (#2630), ATG12 (#4180), ATG16L1 (#8089), Beclin 1 (#3738), β-actin (#4970), CTSD (#2284), LC3B (#2775), p62 (#5114), p38α (#9217), p38β (#2339), phospho-p38α (pThr-180/pThr-182; #9216), phospho-hsp27 (pSer-82; #2406), phospho-MK2 (pThr-222; #3316), Rab7 (#95746), and Rab9 (#5118) were purchased from Cell Signaling Technology (Danvers, MA). Recombinant GST-PIKfyve (full-length, #11-118) was obtained from Carna Biosciences (Natick, MA); and anti-PI(3,5)P2 (Z-P035) was obtained from Echelon Biosciences (Salt Lake City, UT). Anti-tubulin (RE11250C100) was obtained from BioVendor (Candler, NC). Anti-LAMP1 (H4A3) was purchased from Developmental Studies Hybridoma Bank at the University of Iowa (Iowa City, IA). Alexa Fluor™ 488, Alexa Fluor™ 568, and HRP-conjugated secondary antibodies to rabbit and mouse IgG were obtained from Molecular Probes^®^ Invitrogen (Carlsbad, CA), DakoCytomation, Sigma-Aldrich (St. Louis, MO), and Thermo Fisher Scientific (Waltham, MA).

### Plasmid constructs

A cDNA encoding p38α was subcloned into pcDNA6 with an N-terminal myc tag (Invitrogen, Carlsbad, CA), and a kinase dead (D168A) mutant was subsequently generated by site-directed mutagenesis. A constitutively active mutant of p38α (D176A/F327S; obtained from Prof. David Engelberg, Hebrew University, Jerusalem) [66] was subcloned into the *Xho*I-*Bam*HI sites of the pEGFP-C1 vector (Clontech, Mountain View, CA), and the corresponding SB203580-resistant mutant (T106M) was generated by site-directed mutagenesis. An shRNA to human *beclin 1* (5’-1206-GATTGAAGACACAGGAGGC-1225-3’) was cloned into the *Bgl*II/*Xho*I sites of pSuper.retro.puro, transfected in DU145 cells and selected for stable cells using puromycin. A plasmid encoding EGFP-LC3 was provided by Prof. A. Thorburn (University of Colorado Health Sciences Center, Denver, CO) [38].

Labeling of early endosomes was performed by transfecting cells with EGFP-Rab5 (kindly provided by Prof. M. Seabra, Imperial College, UK) [67]. Dominant-negative Rab5 (N133I) was subsequently generated by site-directed mutagenesis. Labeling of LEs was performed by transfecting cells with EGFP-Rab7 or EGFP-Rab9 (provided by Dr B. V. Deurs, University of Copenhagen, Denmark, and Prof. S. Pfeffer, Stanford University, Palo Alto, CA) [68, 69]. Dominant-negative Rab7 (T22N) and Rab9 (S21N) mutants were generated by site-directed mutagenesis. For certain co-labeling experiments, the Rab7 and Rab9 markers were also subcloned into pmCherry-C1 in order to generate mCherry fusion proteins. Finally, lysosomes were labeled using LAMP1-mGFP (provided by Dr E. Dell’Angelica, UCLA, CA) [70].

In order to visualize the generation of PI(3)P, the PX domain of p40Phox (aa 1-149) was cloned by RT-PCR (forward: 5’-GGTACCGAATGGCTGTGGCCCAGCAGC-3’; reverse: 5’-CTCGAGGCGGATGGCCTGGGGCACC-3’) into the *Kpn*I/*Xho*I sites of pcDNA3, in frame with a C-terminal EGFP tag (*Xho*I/*Xba*I) [37]. The PX mutant R57Q, which does not bind to PI(3)P, was subsequently generated by site-directed mutagenesis.

The pLOC lentiviral expression vector was obtained from The University of Texas MD Anderson Cancer Center (UTMDACC) Functional Genomics Core (FGC). Full-length ATG5 was cloned into *Bam*HI/*Nhe*I sites of untagged pLOC and pLOC-HA. Wild-type, kinase dead (D168A) and constitutively active (D176A/F327S) p38α mutants were cloned into the same sites.

### Cell culture and transfections

All cancer cell lines and MEFs were grown in RPMI-1640 or DMEM, supplemented with 5% fetal bovine serum (Atlanta Biologicals, Norcross, GA), 5% Fetalplex (Gemini Bio-products, West Sacramento, CA), 1% penicillin-streptomycin (100 units/mL) and 2 mM Glutamine. Cells were maintained at 37 °C in humidified air containing 5% CO_2_ and were routinely passaged every 3 days. For all transient transfections, with the exception of EGFP-LC3, cells were transfected with 1 µg/mL plasmid DNA using Fugene HD transfection reagent (Roche Diagnostics, Indianapolis, IN).

### Lentiviral transduction and stable cell line generation

HEK293T cells were co-transfected with pLOC along with psPAX2 and pHCMV-G lentivirus packaging plasmids. psPAX2 was a gift from Dr Didier Trono (Addgene plasmid #12260). Approximately 48 h following transfection, the medium was collected and 6 μL of sterile hexadimethrine bromide (5 μg/μL; Sigma-Aldrich #H9268) was added. The collected medium was then filtered through a 0.45-μm PVDF syringe filter and incubated with target cells overnight. Transduction efficiency was evaluated by GFP expression and/or immunoblotting.

### Cell proliferation, endolysosomal volume, and apoptosis Assays

To measure cell proliferation, cells were labeled with CFDA-SE (10 µM; Molecular Probes) for 15 min. Excess CFDA-SE was removed by washing the cells three times with culture media at 25 °C. Labeled cells were then plated in 12-well dishes, treated with SB203580 for several days, and analyzed by flow cytometry (Cytomics FC 500, Beckman-Coulter) for the residual fluorescence in dividing cells. To measure endolysosomal volume, cells were treated for 24–48 h with DMSO (vehicle control) or SB203580, trypsinized, labeled with LysoTracker^TM^ Green DND-26 (50 nM; Molecular Probes) for 60 min at 37 °C, and analyzed by flow cytometry. Apoptosis was assessed by Annexin V/propidium iodide (PI) staining, as previously described [71].

### Vacuolation assays and transmission electron microscopy

To determine the effects of various kinase inhibitors on cytoplasmic vacuolation, DU145 prostate cancer cells were treated with 50 µM of SB203580 or SB202190 (p38), SP600125 (JNKs), PD98059 (ERKs), LY294002 (PI3 kinases), or rapamycin (mTOR). In addition, cells were treated with SB203580 (50 µM) for 24 h in the presence or absence of Baf A_1_ (125 nM). The cells were then examined by phase-contrast light microscopy, and the percentage of vacuolated cells was determined after counting at least 200 cells from three random fields. Each experiment was performed at least three times, and each data point represents mean ± SEM. In experiments performed later in the project, cells (>1000) were analyzed using ImageJ software and a macro developed in-house to determine both vacuole counts and total vacuolated area per cell. For transmission electron microscopy, cells were cultured in 12-well dishes, treated with SB203580 for 24 h, fixed with 1.5% buffered glutaraldehyde and 1% formaldehyde, post-fixed in 2% CaCo-buffered osmium tetroxide-0.8% potassium ferricyanide, embedded in EPON™ resin, sectioned, and analyzed using a Philips EM 208 transmission electron microscope.

### Long-lived protein degradation (LLPD) assay

0.5 × 10^6^–1.0 × 10^6^ cells were plated in duplicate in 6-well plates for each time point, including *t* = 0 h. The next day cells were replenished with fresh medium containing 0.2 μCi/mL L-valine [^14^C(U)] (MC-277, Moravek Biochemicals, Brea, CA) and incubated overnight, after which the hot medium was removed and the cells were washed 3× with Hank’s buffered saline solution (HBSS). The radiolabeled cells were then incubated for 1 h with fresh medium containing 10 mM cold L-valine to prevent reincorporation of liberated L-valine due to rapid protein turnover of short-lived proteins; and *t* = 0 h controls were harvested and precipitated with 10% Trichloroacetic Acid (TCA) to obtain both soluble and insoluble fractions by centrifugation, the latter of which were dissolved in 1 mL 0.2 M NaOH. Counts per minute (CPMs) were then determined for both the soluble and insoluble fractions (100 µL) using a scintillation counter, and the values were multiplied by the 1:10 dilution factor and summed to obtain the total radioactivity (*T*).

In parallel, the remainder of the [^14^C(U)]-labeled cells were washed twice with HBSS and incubated with medium containing 10 mM of cold L-valine and one of the following treatments: dimethyl sulfoxide (DMSO), 3-methyladenine (3-MA; 10 mM), HBSS + DMSO, HBSS + 3-MA, SB203580 (50 µM), or SB203580 + 3-MA. At each time point, 50 μL of media from each well were collected and TCA precipitated with 50 μL of 20% TCA. The CPMs of the soluble fraction were then assayed, as before, and the radioactivity released into the medium (*M*) was calculated by multiplying the measured CPMs by the 1:40 dilution factor. This was repeated at each time point for each treatment; and the fraction of protein degradation (*D*) was calculated as the fraction of radioactivity released into the medium, i.e., the corrected CPMs from each time point (*M*) were divided by the total radioactivity (*T*): (*M*)/(*T*) = (*D*). To isolate protein degradation specific to macroautophagy, the fraction of protein degradation observed in the presence of 3-MA (*D*_*3-MA*_) was subtracted from that observed in its absence: (*D*_DMSO, HBSS, or SB203580_) – (*D*_3-MA_) × 100 = % of 3-MA sensitive protein degradation.

### PIKfyve inhibition assay

Recombinant PIKfyve (Carna Biosciences, #11-118) was incubated with inhibitors (SB203580, SB202190, or YM201636) in kinase assay buffer (50 mM HEPES pH 7.5, 50 mM NaCl, 20 mM MgCl_2_, 0.5 mM EGTA, 0.04% Triton X-100, 25 µg/mL BSA, 0.05 mM DTT) for 30 min at room temperature (RT). Kinase reactions were initiated by adding 5 µL of a mixture of PI(3)P, phosphatidylserine (PS), and ATP. After 2 h, 5 µL of ADP Glo Reagent (Promega), supplemented with 0.01% Triton X-100, was added and incubated for an additional 40 min. Finally, Kinase Detection Reagent (10 µL) was added, incubated for an additional 30 min, and each sample assayed for luminescence. Final assay concentrations: PIKfyve (1 ng/µL), PI(3)P (50 µM), PS (400 µM), ATP (50 µM), inhibitor (0–2000 nM), and DMSO (0.02%).

### Immunofluorescence and western blotting

Cells grown on coverslips were fixed in 4% paraformaldehyde containing 1% sucrose for 15 min at RT; washed 3X in PBS; permeabilized with 1% Triton X-100 for 10 min at RT; and blocked with Background Sniper (Biocare Medical, #BS966) for 20 min at RT, or overnight in PBS containing 10% goat serum. The permeabilized cells were then stained with primary antibodies (1:50) to either Rab7 and/or Rab9 overnight at 4 °C, or LAMP1 for 2 h at RT; washed three times in PBS; and incubated with Alexa Fluor™ 488 or Alexa Fluor™ 568 secondary antibodies (1:500) for 1 h at RT. Finally, cells were washed with PBS (3×) and PBST (1×); counterstained with DAPI; rinsed in PBS; mounted in Prolong Gold Anti-fade mounting media (Invitrogen #9071); and analyzed by confocal microscopy (Zeiss LSM880 with Airyscan). Fluorescence live cell imaging on EGFP and mCherry-transfected cells was performed using a Nikon Eclipse TE2000S. Western blotting was performed as previously described [71].

### LysoSensor™ staining and image analysis

DU145 cells were first incubated with DMSO or SB203580 (50 µM) for 24 h, followed by the addition of 2 µM LysoSensor™ Yellow/Blue DND-160 (Thermo Fisher Scientific, Cat # L7525) for 10 min. Cells were then evaluated at the MDACC Advanced Microscopy and Cell Imaging Core using a Zeiss LSM880 with Airyscan confocal microscope, using a Zeiss 40× c-apo (N.A. 1.2) water immersion lens; images were collected using an excitation wavelength of 355 nm (Coherent) and emission wavelengths in the yellow (510–641 nm) and blue (404–456 nm) channels, as previously described [72]. Image analysis was performed using Imaris (Andor/Oxford Instruments) software (version 9.9) Surfaces module; fluorescence intensities and lysosome/vacuole volumes were obtained for each and exported to GraphPad Prism software to calculate and graph their corresponding yellow/blue ratios.

### Statistical analyses

Unless otherwise indicated, all experiments were performed at least three times and statistical significance was determined in GraphPad Prism software using either a one- or two-way ANOVA with Student–Newman–Kuel’s or Šídák’s post hoc analyses, respectively.

### Supplementary information

Supplementary Legends

Supplementary Figures

Whole uncropped blots

## Data Availability

The datasets generated during and/or analyzed during the current study are available from the corresponding author on reasonable request.
