# Supplementary material for: Unexpected inhibition of the lipid kinase PIKfyve reveals an epistatic role for p38 MAPKs in endolysosomal fission and volume control
Source: Cell Death Dis. 2024 Jan 22;15(1):80. doi: 10.1038/s41419-024-06423-0 (PMC10803372; doi:10.1038/s41419-024-06423-0)
Supplement: Supplementary file 1 — Supplementary Legends [file 41419_2024_6423_MOESM1_ESM.docx]

**Supplemental Figure Legends**

**fig S1. p38 MAPK inhibitors, but not JNK, ERK, mTOR, or PI3K inhibitors, induce vacuolation.** *(A)* Various cancer cell lines were treated with DMSO (control), SB203580 (50 µM), or SB202190 (50 µM) for 24 h and assessed for vacuolation by phase-contrast microscopy. *(B)* DU145 cells were treated with various kinase inhibitors (50 µM), including SB203580, SP600125, PD98059, rapamycin, and LY294002, and assessed for vacuolation and cell death. *(C)* DU145 cells were treated with SB203580 (50 µM) for 24 h and then washed and replaced with fresh media ± SB203580 for 4-24 h. At each time point following the washout, cells were examined for vacuolation by phase-contrast microscopy (*see* also Fig. 1D). *(D and E)* Concentration-dependent inhibition of HSP27 phosphoryla­tion by SB203580 (0 - 100 µM) was determined by western blotting, and individual bands were scanned, quantified with ImageJ software, and plotted as the percent of p-HSP27 inhibited (*see* also Fig. 1E). At each SB203580 concentration, the percentages of vacuolated cells were plotted against the losses in HSP27 phosphorylation and analyzed by linear regression (R^2^ = 0.82). *(F)* DU145 cells were treated with SB202190 (50 µM) for 24 h and evaluated for its ability to inhibit p38 MAPK-dependent phosphorylation of HSP27.

**fig S2. SB203580 does not induce the formation of double-bilayered autophagosomes and does not stimulate long-lived protein degradation.** *(A)* DU145 cells were treated with *(i)* DMSO or *(ii-iv)* SB203580 (50 µM) for 24 h and analyzed by transmission electron microscopy. Whole cells in panels *i* and *ii* were magnified 4,400X and 2,800X, respectively (bars = 2 µm); and both large translucent and partially filled vacuoles were visible in SB203580-treated cells. Representative vacuoles (panels *iii* and *iv*) were magnified 28,000X and 44,000X, respectively (bars = 500 nm and 200 nm) and clearly contained partially digested material. *(B)* DU145, A549, HCT116, and HT-29 cancer cell lines were labeled with [^14^C(U)]-L-Valine (0.2 µCi/mL media), starved or exposed to SB203580, in the absence or presence of 3-MA, and assayed for LLPD by scintillation counting, as described in the methods.

**fig S3. Rab7 mediates SB203580-induced vacuolation.** *(A)* DU145 cells were co-transfected with various combinations of EGFP-LAMP1 and mCherry-Rab7. The cells were then treated with DMSO (control) or SB203580 (50 µM) for 24 h and examined for colocalization with the vacuoles and/or one another. *(B)* DU145 cells, treated for 24 h with SB203580 (50 µM), were evaluated by immunofluorescence microscopy for endogenous Rab7 (green) and Rab9 (red) (supplemental images for Fig. 3B). Field #2 illustrates Rab7 labeling of the larger vacuoles, whereas in Field #3 smaller vacuoles are labeled with either Rab7 or Rab9. *(C)* DU145 cells were similarly transfected with either EGFP (empty vector), a dominant-negative Rab5(N133I) mutant, a constitutively active Rab7(Q67L) mutant, or a Rab9(S21N) dominant-negative mutant. Cells were then exposed to DMSO or SB203580 (50 µM), and the number of vacuoles was counted in at least 50 cells by fluorescence microscopy. Each experiment was performed in triplicate, and each data point represents mean ± SEM (*see* also Fig. 3C).

**fig S4. SB203580-induced vacuolation is reversible and results from LEL swelling.** *(A)* Representative images for Fig. 4H. *(B)* DU145 cells were exposed to SB203580 (50 µM) for 24 h, followed by the addition of DMSO or Bafilomycin A1 (125 nM) for an additional 24 h. Vacuolation was then evaluated by phase-contrast microscopy and quantified using ImageJ software. *(C)* DU145 cells were treated with SB203580 (50 µM) for 24 h and then washed and replaced with fresh media ± SB203580 (50 µM) for 4-24 h. At each time point following the washout, cells were stained with LysoTracker^TM^ Green and analyzed by flow cytometry for acidification. *(D)* Following a 3 h washout ± SB203580 (50 µM), changes in vacuolation were examined by immunofluorescence microscopy using an anti-LAMP1 antibody (panels *i,ii*: 600X) and by transmission electron microscopy (panel *iii*: 4,400X, bar = 2 µm; panel *iv*: 28,000X, bar = 500 nm). Following a 1 h washout of SB203580, the large cytoplasmic vacuoles began to collapse, flatten out, and undergo fission (panel *v*: 5,600X, bar = 2 µm; panel *vi*: 14,000X, bar = 500 nm). *(E,F)* DU145 cells were treated with SB203580 (50 µM) for 24 h, in the presence or absence of sorbitol (1 mM), and evaluated for vacuolation by phase-contrast microscopy. *(G)* Cartoon illustrating various ion channels that maintain normal osmotic balance in LELs. *(H,I)* DU145 cells were treated with SB203580 (50 µM) for 24 h, in the presence or absence of a Na+-H+ exchanger inhibitor (Zonaporide, 25-500 µM), or an CLC chloride channel inhibitor (4,4′-Diisothiocyanatostilbene-2,2′-disulfonic acid, *i.e.* DIDS, 1-4 mM). The cells were then evaluated for vacuole formation by flow cytometry (LysoTracker^TM^ Green) or phase-contrast microscopy.

**fig S5. The p38 MAPK inhibitor BIRB-796 enhances vacuolation induced by PIKfyve inhibitors.** *(A-C)* Wild-type DU145 cells were treated with YM201636 (500 nM) or apilimod (20 nM), in the presence or absence of BIRB-796 (50 µM) for 24 h and evaluated for vacuolation by phase-contrast microscopy and ImageJ analysis software.
