# Supplementary figures and images for: Unexpected inhibition of the lipid kinase PIKfyve reveals an epistatic role for p38 MAPKs in endolysosomal fission and volume control
Source: Cell Death Dis. 2024 Jan 22;15(1):80. doi: 10.1038/s41419-024-06423-0 (PMC10803372; doi:10.1038/s41419-024-06423-0)

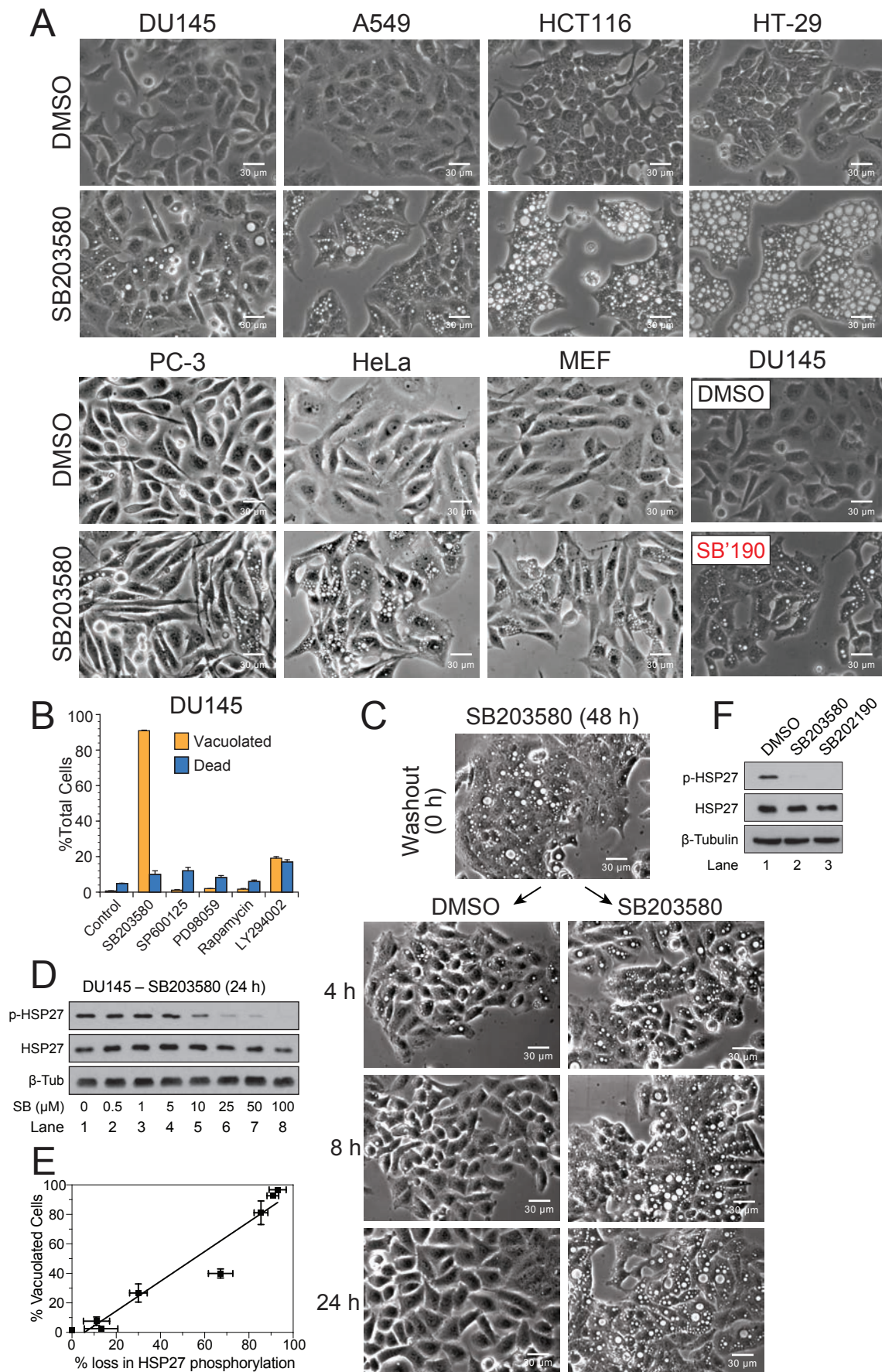

A

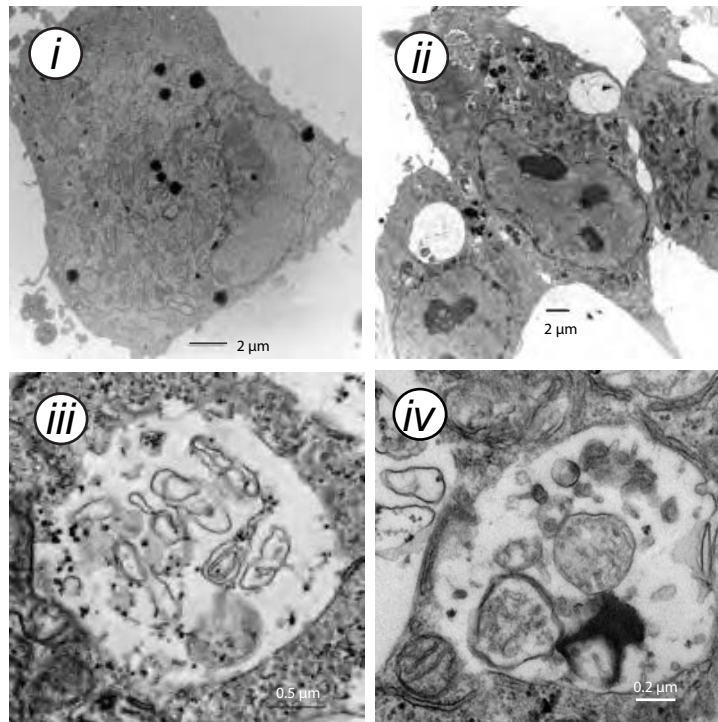

B

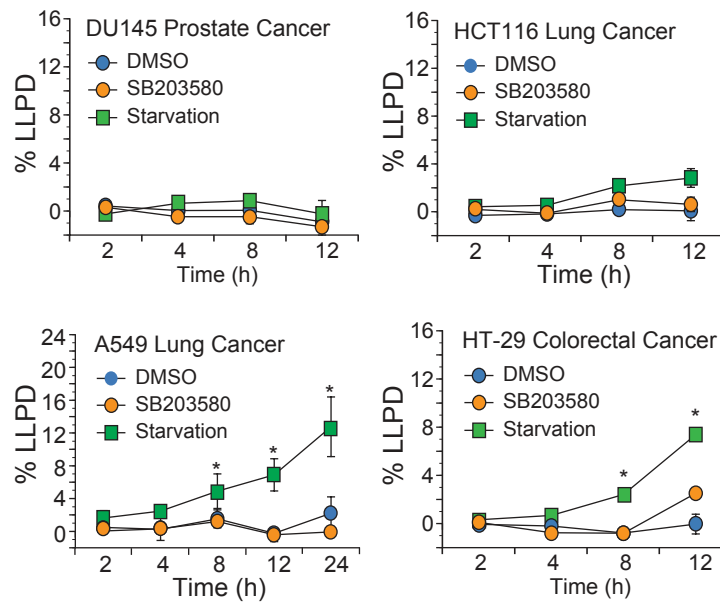

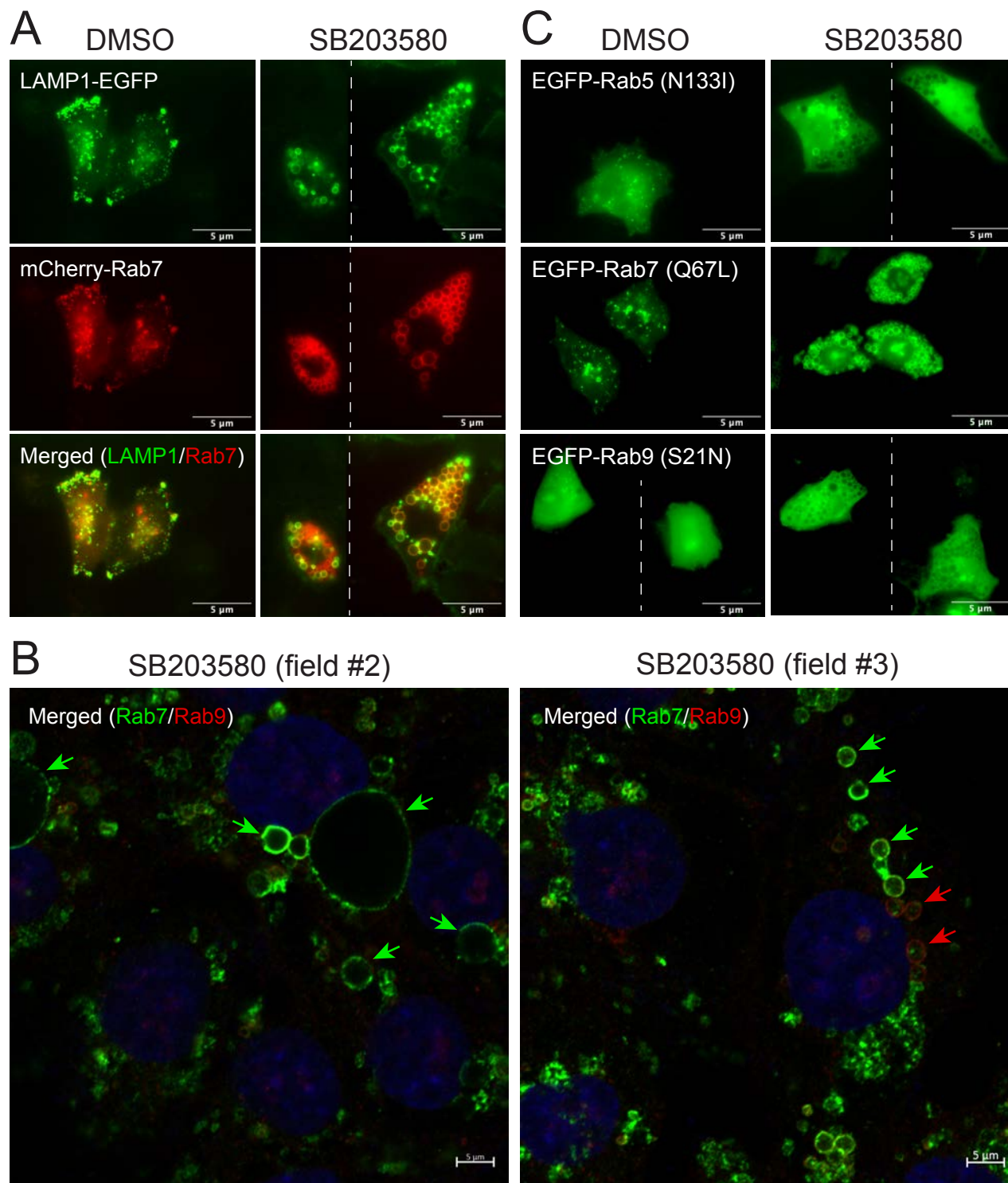

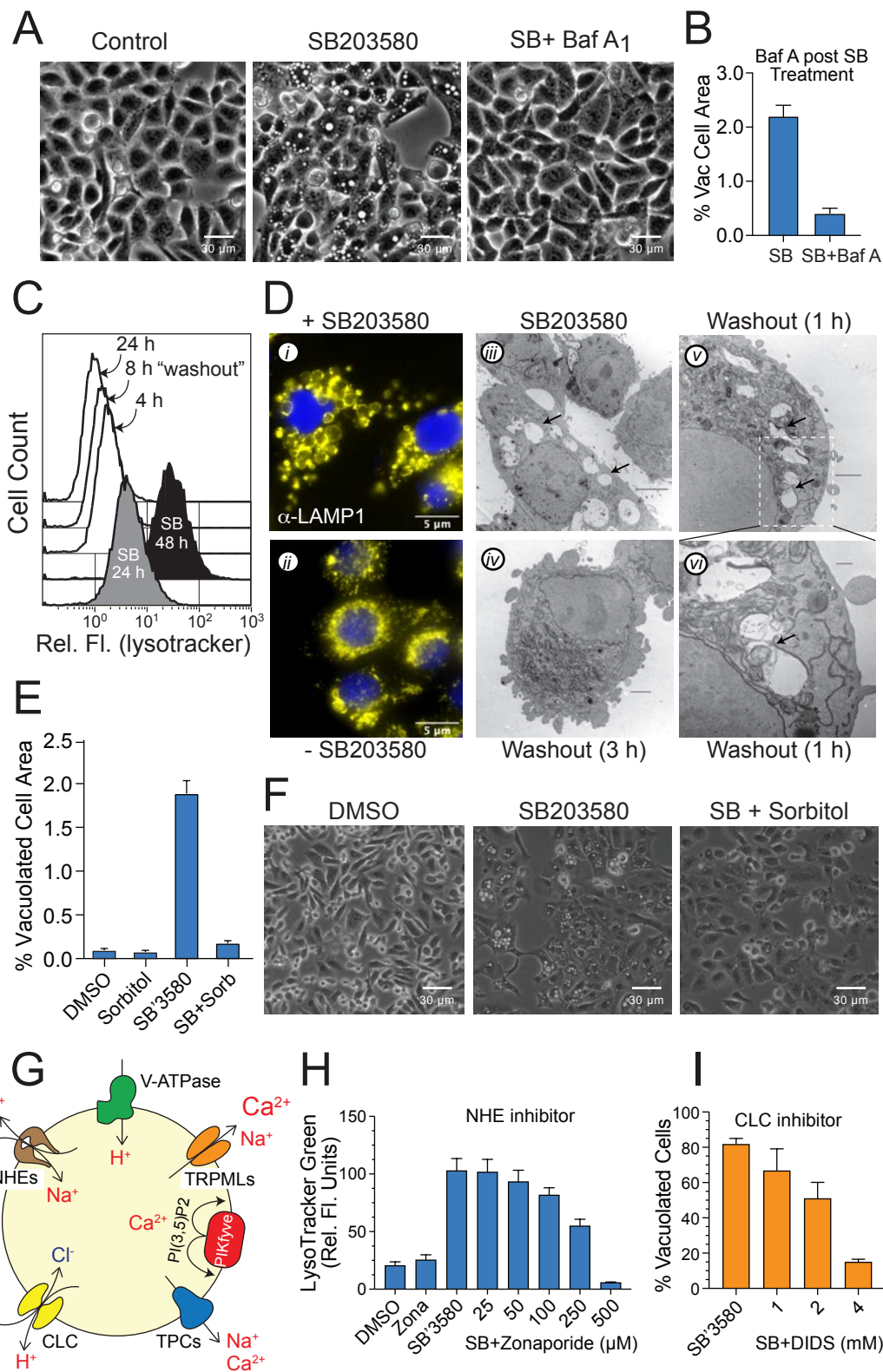

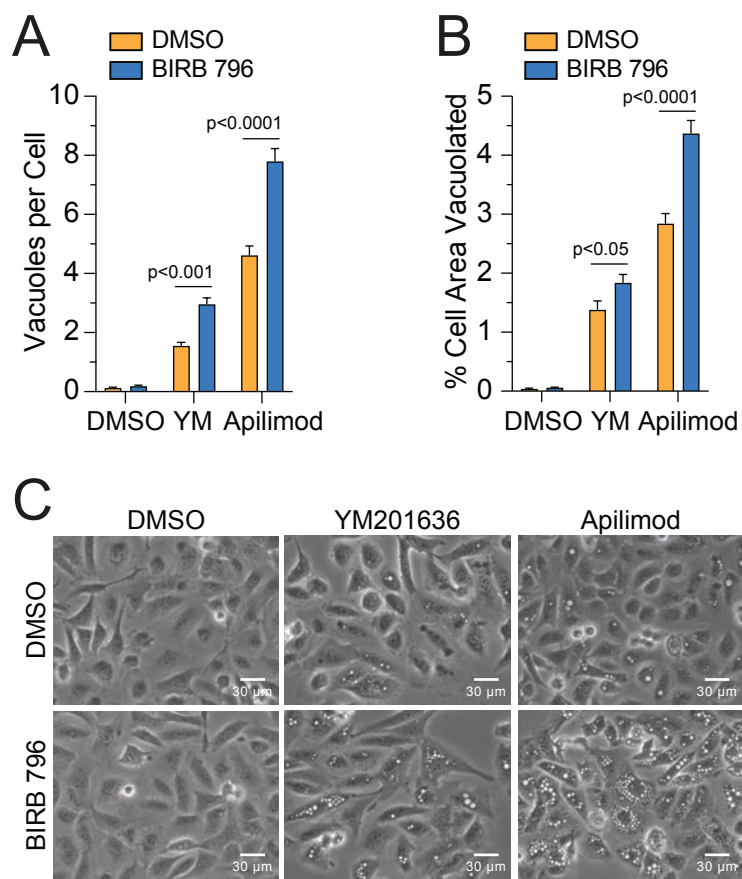

Supplement: Supplementary file 2 — Supplementary Figures [file 41419_2024_6423_MOESM2_ESM.pdf]
