# Supplementary material for: Unexpected inhibition of the lipid kinase PIKfyve reveals an epistatic role for p38 MAPKs in endolysosomal fission and volume control
Source: Cell Death Dis. 2024 Jan 22;15(1):80. doi: 10.1038/s41419-024-06423-0 (PMC10803372; doi:10.1038/s41419-024-06423-0)
Supplement: Supplementary file 3 — Whole uncropped blots [file 41419_2024_6423_MOESM3_ESM.pdf]

Fig 1D

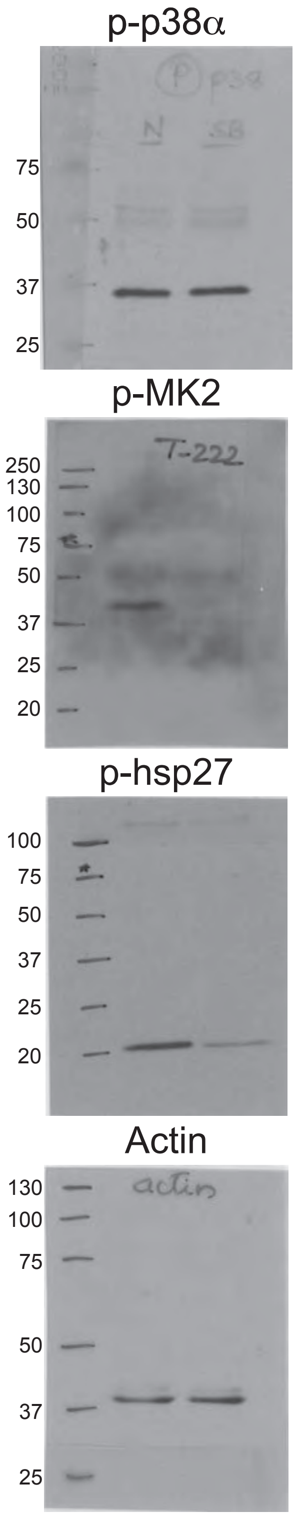

Fig 1G

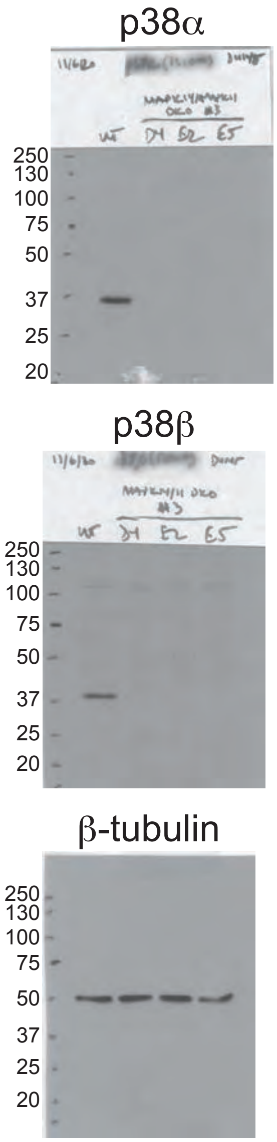

fig S1E

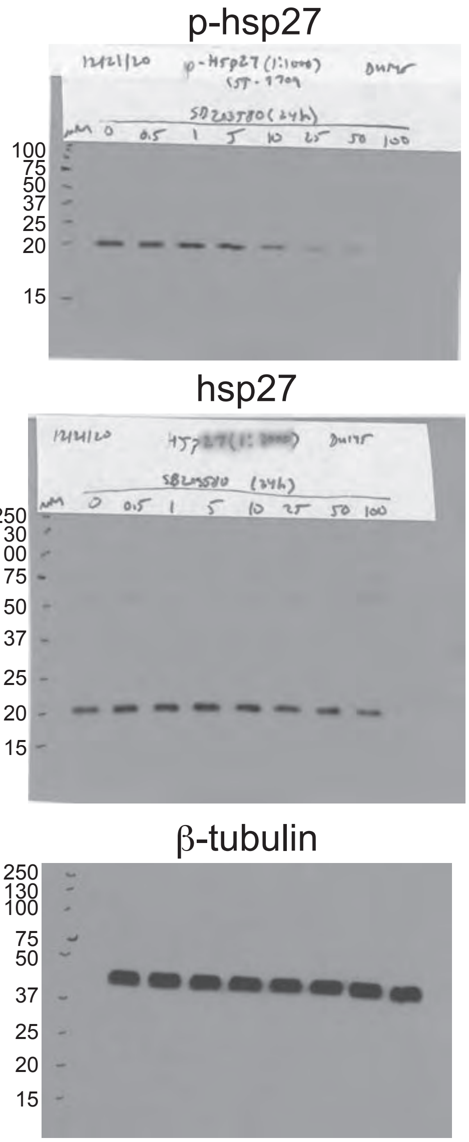

Fig 2E

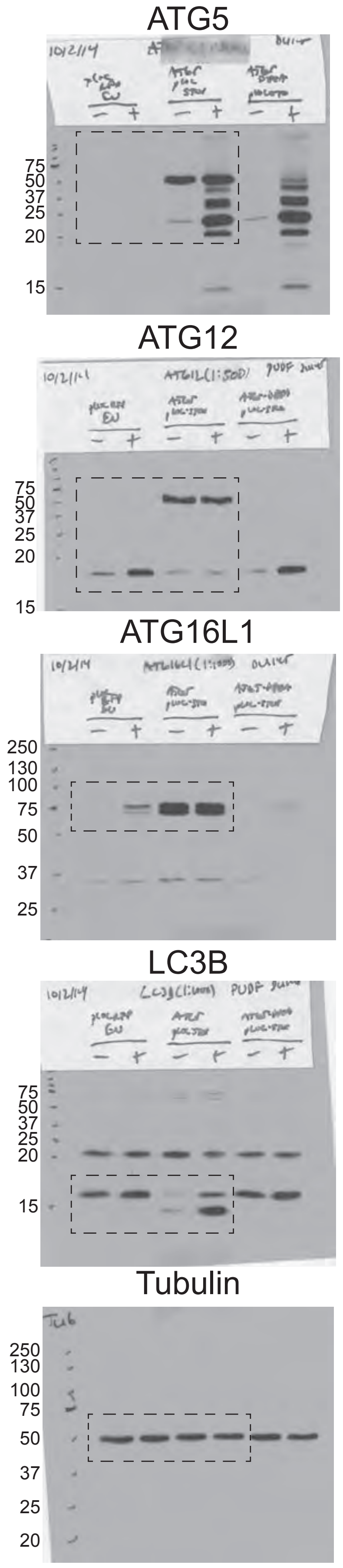

Fig 2B

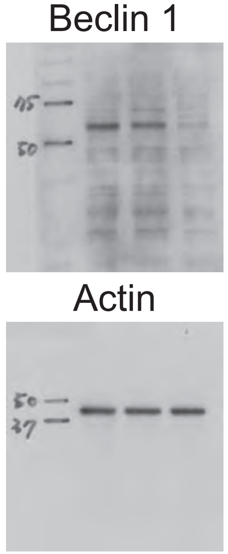

Fig 4A

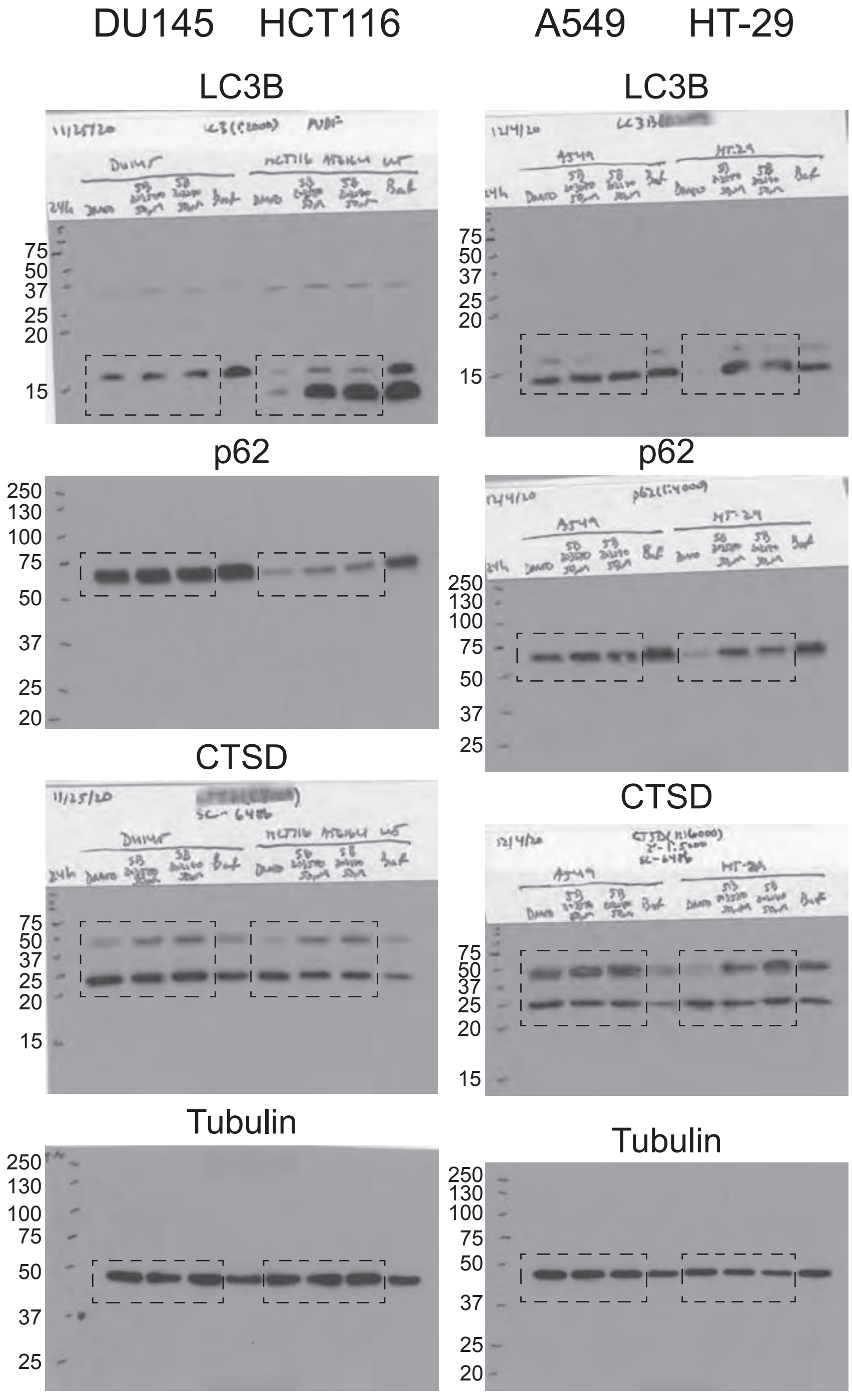

DU145 HCT116
A549 HT-29
LC3B
p62
CTSD
Tubulin
75
50
37
25
20
15
250
130
100
